# Aberrant Expression of Long Noncoding RNAs in Autistic Brain

**DOI:** 10.1007/s12031-012-9880-8

**Published:** 2012-09-05

**Authors:** Mark N. Ziats, Owen M. Rennert

**Affiliations:** 1 Laboratory of Clinical and Developmental Genomics, National Institute of Child Health and Human Development, National Institutes of Health, 49 Convent Drive, Building 49, Room 2C08, Bethesda, MD 20814 USA; 2 Laboratory of Clinical and Developmental Genomics, National Institute of Child Health and Human Development, National Institutes of Health, 49 Convent Drive, Building 49, Room 2A08, Bethesda, MD 20814 USA; 3 Baylor College of Medicine MSTP, Houston, TX USA; 4 NIH-University of Cambridge Biomedical Scholars Program, Cambridge, UK

**Keywords:** Noncoding RNA, Long noncoding RNA, Genomics, Autistic disorder, Gene expression

## Abstract

The autism spectrum disorders (ASD) have a significant hereditary component, but the implicated genetic loci are heterogeneous and complex. Consequently, there is a gap in understanding how diverse genomic aberrations all result in one clinical ASD phenotype. Gene expression studies from autism brain tissue have demonstrated that aberrantly expressed protein-coding genes may converge onto common molecular pathways, potentially reconciling the strong heritability and shared clinical phenotypes with the genomic heterogeneity of the disorder. However, the regulation of gene expression is extremely complex and governed by many mechanisms, including noncoding RNAs. Yet no study in ASD brain tissue has assessed for changes in regulatory long noncoding RNAs (lncRNAs), which represent a large proportion of the human transcriptome, and actively modulate mRNA expression. To assess if aberrant expression of lncRNAs may play a role in the molecular pathogenesis of ASD, we profiled over 33,000 annotated lncRNAs and 30,000 mRNA transcripts from postmortem brain tissue of autistic and control prefrontal cortex and cerebellum by microarray. We detected over 200 differentially expressed lncRNAs in ASD, which were enriched for genomic regions containing genes related to neurodevelopment and psychiatric disease. Additionally, comparison of differences in expression of mRNAs between prefrontal cortex and cerebellum within individual donors showed ASD brains had more transcriptional homogeneity. Moreover, this was also true of the lncRNA transcriptome. Our results suggest that further investigation of lncRNA expression in autistic brain may further elucidate the molecular pathogenesis of this disorder.

**Electronic supplementary material:**

The online version of this article (doi:10.1007/s12031-012-9880-8) contains supplementary material, which is available to authorized users.

## Introduction

The autism spectrum disorders (ASD) are one of the most heritable of the common neuropsychiatric conditions (Miles 2011). However, there are hundreds of implicated genomic loci with heterogeneous functions (Holt and Monaco 2011). As a result, there is difficulty in understanding how this diverse genetic susceptibility translates to a common clinical phenotype. Multiple gene expression studies in postmortem autism brain tissue suggest that aberrant processing of the mRNA transcriptome in autistic brains may provide a mechanistic convergence between this diverse genetic heritability and disruption of fundamental neurodevelopmental pathways leading to the common ASD phenotype (Geschwind 2008). For example, three transcription studies in autism brain tissue have demonstrated convergence of differentially expressed genes in autism brains on immune and synapse formation pathways (Purcell et al. 2001; Garbett et al. 2008; Voineagu et al. 2011). Similarly, gene expression studies in other tissue types from autism patients suggest a convergence of differentially expressed mRNAs on similar fundamental molecular pathways (Lintas et al. 2012; Voineagu 2012). However, the regulation (and consequently dysregulation) of gene expression is a very complex process, resulting from an interplay of DNA sequence variation, chromatin and epigenetic modifications, protein transcription factors, and regulatory noncoding RNAs.

While transcriptome studies in autistic brain samples have demonstrated that aberrant expression of mRNA transcripts may represent a convergence of the heterogeneous genomics of ASD, none of these studies have concurrently assessed the regulatory RNAs that may underlie aberrant mRNA expression. Three studies in lymphoblast cell lines from autism patients have shown that miRNAs are abnormal in autism (Talebizadeh et al. 2008; Sarachana et al. 2010; Seno et al. 2011), and Abu-Elneel et al. demonstrated differential expression of miRNAs in autistic cerebellum (Abu-Elneel et al. 2008). However, a novel class of regulatory RNAs, long noncoding RNAs (lncRNAs), has recently been implicated in a number of fundamental gene regulatory events, but their role in autism molecular pathogenesis remains unknown.

Long noncoding RNAs are defined as RNAs greater than 200 nucleotides in length (as compared to ~21–23 nucleotide length of miRNAs), which do not encode for protein. While originally thought to be “transcriptional noise,” long noncoding RNAs have been shown to be involved in major mechanisms of gene expression regulation, such as targeting transcription factors, initiating chromatin remodeling, directing methylation complexes, and blocking nearby transcription (Ponting et al. 2009). Moreover, pervasive transcription of lncRNAs has been demonstrated to occur in both a temporally and spatially regulated manner during development (Amaral and Mattick 2008), with the central nervous system displaying the greatest abundance of transcribed lncRNAs (Mercer et al. 2008). So, while there have been multiple studies at the transcriptome level in autism demonstrating aberrant expression of mRNAs, none have assessed long noncoding RNAs, which are increasingly recognized as a fundamental to gene expression regulation.

Therefore, the purpose of this study was to determine if dysregulated expression of lncRNAs might play a role in the molecular pathogenesis of ASD. To do so, we profiled over 33,000 annotated lncRNAs in ASD patient postmortem brain tissue (prefrontal cortex and cerebellum) using microarrays. In parallel, we also assessed for transcriptional differences in all known protein-coding mRNAs. We identified over 200 differentially expressed lncRNAs, which were oriented in or around protein-coding loci strongly enriched for brain development genes. Moreover, we discovered that the previously reported homogeneity of mRNA transcription within autism brains is also observed within the lncRNA component of the transcriptome.

## Materials and Methods

### Brain Tissue

Human postmortem brain tissue was obtained from the NICHD Brain and Tissue Bank for Developmental Disorders at the University of Maryland, Baltimore, MD, USA. The collection protocol at the University of Maryland, Baltimore was reviewed and approved by the Institutional Review Board of the University of Maryland, Baltimore. The clinical characteristics of the patients from whom tissue was derived are shown in the Supplementary Data—Additional File 1, Table 1. Both the prefrontal cortex and cerebellum samples were obtained from two autism patients and two age- and sex-matched controls (total biological replicates of *n* = 4 for autism and controls).

### RNA Isolation and Quality Control

Total RNA was extracted by homogenizing samples in TRIzol® Reagent (Invitrogen) according to the manufacturer’s protocol. RNA quantity was measured by NanoDrop ND-1000. Agilent Bioanalyzer 2100 was used to assess RNA integrity for each sample (Supplementary Data—Additional File 1, Table 1).

### lncRNA Microarray

ArrayStar, Inc (Rockville, MD, USA) Human lncRNA Microarray V2.0 was used and run by the service provider. The array contains 33,045 lncRNAs and 30,215 protein-coding transcripts. The lncRNAs were manually collected from the most authoritative databases such as RefSeq, UCSC knowngenes, Ensembl, and manually curated lncRNA literature sources (Supplementary Data—Additional File 1, Table 2). The mRNAs were obtained from RefSeq (March 2011). Each transcript was represented by a specific exon or splice junction probe. Positive probes for housekeeping genes and negative control probes (i.e., scramble sequences) were also printed onto the array for hybridization quality control.

### Microarray Labeling, Hybridization, and Scanning

Sample labeling and array hybridization were performed according to the Agilent One-Color Microarray-Based Gene Expression Analysis protocol (Agilent Technology) with minor modifications. Briefly, mRNA was purified from 1 μg total RNA after removal of rRNA (mRNA-ONLY™ Eukaryotic mRNA Isolation Kit, Epicentre). Then, each sample was amplified and transcribed into fluorescent cRNA along the entire length of the transcripts without 3′ bias utilizing a random priming method. The labeled cRNAs were purified by RNAeasy Mini Kit (Qiagen). The concentration and specific activity of the labeled cRNAs (picomole of Cy3/microgram of cRNA) were measured by NanoDrop ND-1000. One microgram of each labeled cRNA was fragmented by adding 11 μl of 10× blocking agent and 2.2 μl of 25× fragmentation buffer, heating the mixture at 60 °C for 30 min, then adding 55 μl of 2× GE hybridization buffer to dilute the labeled cRNA. One hundred microliters of hybridization solution was dispensed into the gasket slide and assembled to the lncRNA expression microarray slide. The slides were incubated for 17 h at 65 °C in an Agilent Hybridization Oven. The hybridized arrays were washed, fixed, and scanned using the Agilent DNA Microarray Scanner (G2505B).

### Microarray Data Processing

Agilent Feature Extraction software (version 10.5.1.1) was used to analyze acquired array images. Quantile normalization and subsequent data processing were performed using the GeneSpring GX v11.5.1 software package (Agilent Technologies). After quantile normalization of the raw data, lncRNAs and mRNAs with at least four out of eight samples flagged as 'present' or 'marginal' were chosen for further analysis. Differentially expressed lncRNAs and mRNAs were identified through fold change filtering. Differentially expressed lncRNAs and mRNAs with statistical significance (as determined by two-tailed Student’s *t* test < 0.05) were identified through volcano plot filtering. All microarray data were deposited into Gene Expression Omnibus (GEO) at the National Center for Biotechnology Information, NIH under Series Number GSE36315.

### qRT-PCR

Five randomly selected lncRNAs from among those showing the greatest fold change were chosen for confirmation via quantitative real-time reverse transcriptase PCR (qRT-PCR). The selected lncRNAs and the primers used for qRT-PCR are shown in Supplementary Data—Additional File 1, Table 3. Five micrograms of total RNA was used for the synthesis of first strand cDNA using the SuperScript III First Strand cDNA Synthesis Kit (Invitrogen). qRT-PCR analysis was performed using ABI prism 7900 (Applied Biosystems) with SYBR Green expression assay system (Applied Biosystems). Normalized, relative gene expression was calculated using standard ∆∆Ct methods using Applied Biosystem RQ Manager Software (v1.2). Each qPCR reaction was run three separate times, with technical triplicates in each reaction.

### In Silico Mapping Analysis

To assess for the potential *cis*-regulatory effects of the identified lncRNAs, we utilized the Genomic Regions Enrichment of Annotations Tool (Mclean et al. 2010). This program takes genomic coordinates as inputs and outputs nearby genes and their ontologies. Default settings were used for analysis on all probes detected as differentially expressed between ASD and Ctrl (both prefrontal cortex and cerebellum), with curated regulatory domains included.

### Gene Ontology Enrichment Analysis

To assess for functional categories that the genes we identified as significantly differentially expressed in ASD implicated, we used the Database for Annotation, Visualization and Integrated Discovery v6.7, accessed at http://david.abcc.ncifcrf.gov/. GO categories were reported as significant only if the *p* value after multiple testing corrections was <0.05.

## Results

**Fig. 1:**
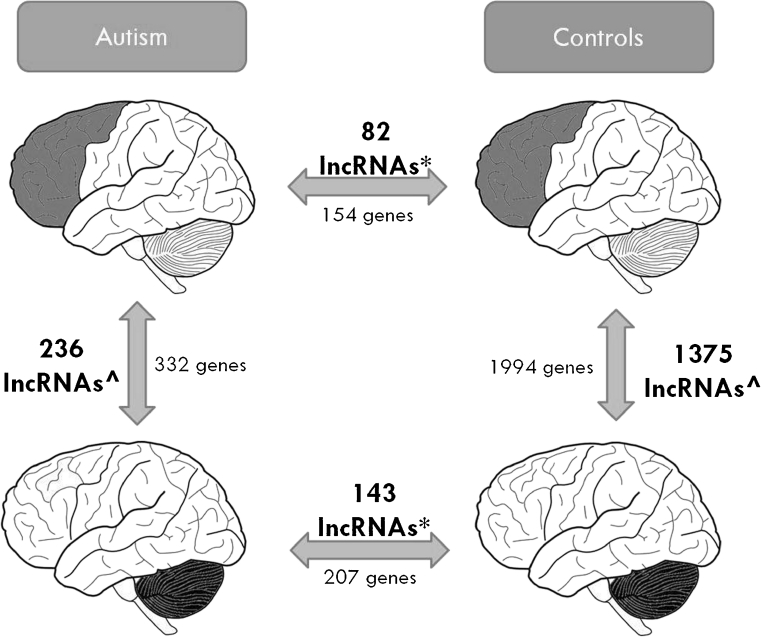
Summary of differentially expressed lncRNAs and mRNAs. *Asterisk*, three shared lncRNAs; *caret,* 99 shared lncRNAs

In total, we detected 222 lncRNAs differentially expressed between ASD and control samples (fold change > 2, *p* < 0.05). Eighty-two of these were unique to the prefrontal cortex, and 143 were unique to the cerebellum (Fig. 1). The majority of differentially expressed lncRNAs in ASD were from intergenic regions (~60 %), antisense to protein-coding loci (~15 %), or within introns of protein-coding genes (~10 %), with the others representing overlapping transcripts from exons or introns in both sense and antisense directions. This distribution was not significantly different from the distribution of all lncRNAs detected by the array (Supplementary Data—Additional File 1, Fig. 1). We confirmed a select number of the most highly differentially expressed lncRNAs between autism and controls by qRT-PCR analysis (Supplementary Data—Additional File 1, Fig. 2).

Almost 50 % of differentially expressed lncRNAs map to within 50 kilobases (kb) of an annotated gene, and greater than 90 % map within 500 kb of a known gene (Supplementary Data—Additional File 1, Fig. 3). Mapping all differentially expressed lncRNAs to the nearest genes identified 381 protein-coding loci under putative *cis*-regulatory control by these lncRNAs. The ontologies of those loci implicated two functions: cerebral cortex cell migration and targets of microRNAs mir-103/107 (Table 1). These results are intriguing given that the prevailing cellular model of autism is a defect in neuronal connectivity (Geschwind and Levitt 2007) and that mir-103/107 has previously been implicated in CNS development (Moncini et al. 2011), Alzheimer’s disease (Nelson and Wang 2010), and schizophrenia (Santarelli et al. 2011). Eleven of these genes near differentially expressed lncRNAs have previously been implicated in ASD, and 18 have previously been shown to exhibit differential expression in ASD brain (Supplementary Data—Additional File 1, Table 4).

**Table 1:** Gene ontology analysis of 381 mRNA loci nearby differentially expressed lncRNAs

Ontology	Term name	Binomial FDR *Q* value	Binomial fold enrichment	Hypergeometric FDR *Q* value	Hypergeometric fold enrichment
GO biological Process	Cerebral cortex cell migration	3.16e − 2	4.68	2.06e − 2	12.82
MSigDB miRNA motifs	Targets of miR-103/miR-107	5.88e − 8	3.18	3.85e − 2	3.30

Ninety of the differentially expressed lncRNAs are oriented in or around a known protein-coding region (i.e., not intergenic). Of these, three are known imprinted loci in humans (C9orf85, SLC4A2, and UBE3A). Interestingly, UBE3A is implicated in the genomic imprinting disorder Angelman syndrome, which shares many features with ASD (Bonati et al. 2007). Surprisingly, however, only 3 of these 90 genes are also differentially expressed (RBM8a, ARL17A, and KLF6), suggesting perhaps more complex mechanisms for many of these lncRNAs than simple *cis*-regulation.

Our array also contained probes for known protein-coding transcripts, of which we detected 355 genes differentially expressed between ASD and controls, which were enriched for the process of alternative splicing (Supplementary Data—Additional File 1, Table 5). This finding is in agreement with a recent large transcriptome study in autistic brains by Voineagu et al., where they demonstrated dysregulated splicing of *A2BP1*-dependent exons in ASD brains using RNA-seq (Voineagu et al. 2011).

Because our samples from the prefrontal cortex and cerebellum were from the same patients, we also had the ability to compare intra-individual differences in expression of both genes and lncRNAs between these regions. We detected almost 2,000 genes differentially expressed in the control prefrontal cortex versus the control cerebellum, which were highly enriched for gene ontology terms related to synaptogenesis (Supplementary Data—Additional File 1, Table 6), but only 322 genes differentially expressed between the ASD prefrontal cortex and cerebellum (Fig. 1). These results are also in agreement with the study by Voineagu and colleagues, where they observed more transcriptional homogeneity in ASD brains (Voineagu et al. 2011). In light of this, then, we were intrigued to find that the number of lncRNAs differentially expressed within control brains was also much greater than lncRNAs differentially expressed within autism brains (1,375 lncRNAs versus 236 lncRNAs, respectively).

## Discussion

While there have been multiple studies of the mRNA transcriptome in ASD, we report here the first assessment of regulatory lncRNAs in autism postmortem brain tissue. We identified lncRNAs that are differentially expressed in ASD brain tissue and showed that they are enriched for genomic loci involved in neurodevelopment and psychiatric disease. Notably, *trans*-regulatory mechanisms of these lncRNAs are likely to be major contributors to their cellular importance. Future studies using knockdown or overexpression techniques in a relevant model system would be a reasonable approach to uncover potential *trans*-regulatory effects.

Furthermore, both the lncRNA and the mRNA transcriptome appear to be more differentially expressed within control brains (between prefrontal cortex and cerebellum) as compared to ASD brains. This finding is particularly interesting in the context of imaging studies of autistic brain, where it has been suggested that anatomically distinct regions of the autistic brain are less specialized from each other than in healthy subjects (Minshew and Keller 2010). It is intriguing to speculate that perhaps less “genomic differentiation” between brain regions in autism underlies these imaging findings.

In summary, these results identify lncRNAs that are aberrantly expressed in autistic brain and suggest that perhaps lncRNAs contribute to dysregulation of protein-coding loci in ASD and/or that a fundamental defect in genome-wide transcriptional regulation—including noncoding regions of the genome—underlies ASD molecular pathology. Future studies will need to replicate and expand these findings in more patient samples, but this initial evidence suggests that the lncRNA component of the transcriptome deserves attention in autism.

## Electronic supplementary materials

Below is the link to the electronic supplementary material.

ESM 1 Contains all supplementary figures and tables (DOC 324 kb)

ESM 2 List and annotation of all differentially expressed lncRNAs (XLS 78 kb)

ESM 3 List and annotation of all differentially expressed mRNAs (XLS 693 kb)
