# Supplementary material for: Aberrant Expression of Long Noncoding RNAs in Autistic Brain
Source: J Mol Neurosci. 2012 Sep 5;49(3):589–93. doi: 10.1007/s12031-012-9880-8 (PMC3566384; doi:10.1007/s12031-012-9880-8)
Supplement: Supplementary file 1 — Contains all supplementary figures and tables (DOC 324 kb) [file 12031_2012_9880_MOESM1_ESM.doc]

Additional File 1

**Aberrant expression of long non-coding RNAs in autistic brain**

Mark N. Ziats and Owen M. Rennert

Supplementary Figures

**Supplementary Figure 1. Distribution of differentially expressed lncRNAs by genomic origin**. All lncRNAs that were detected by the array in our samples were mapped to their genomic origin, and this distribution was compared to the 222 lncRNAs that were differentially expressed in ASD brains (shown below), which was not significantly different (chi-square p-value = 0.33).

**
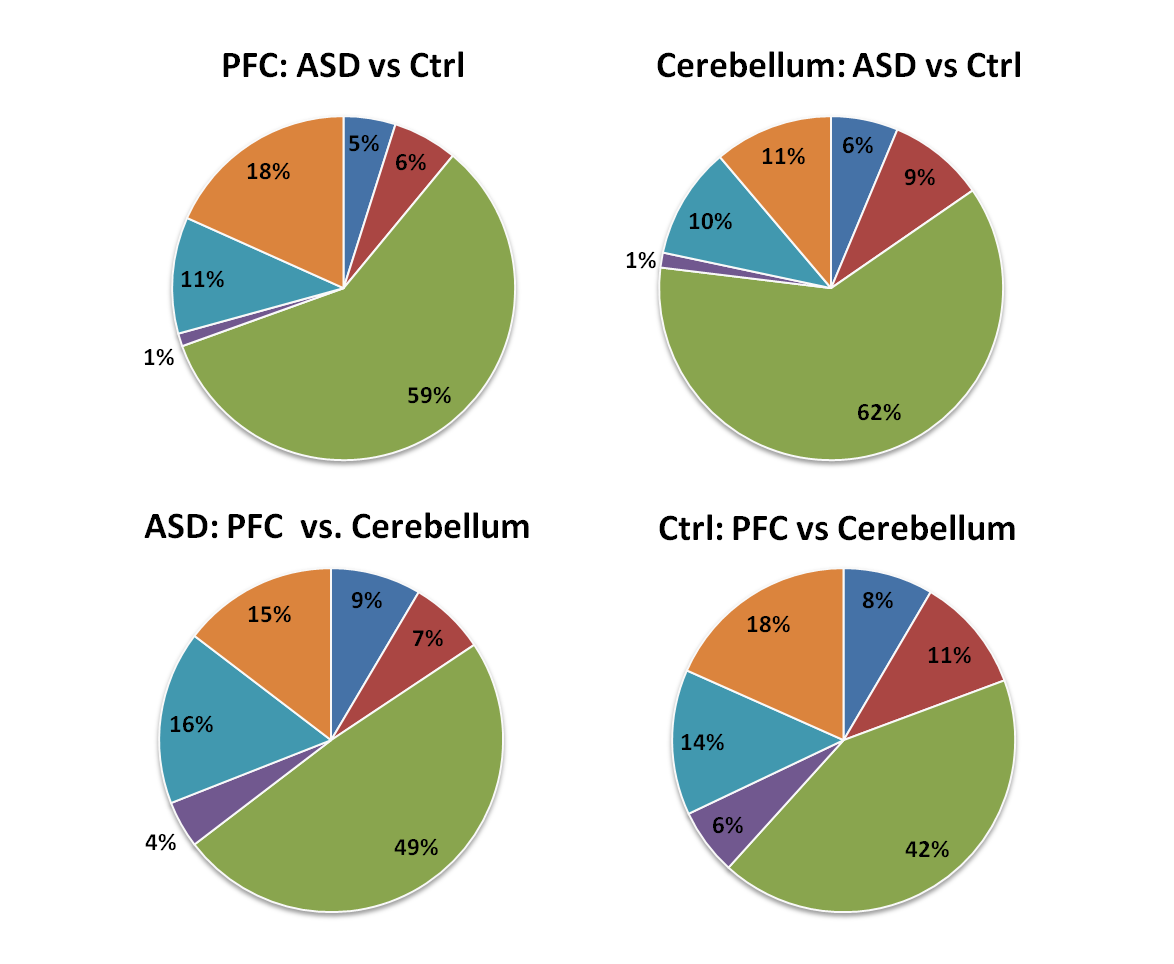

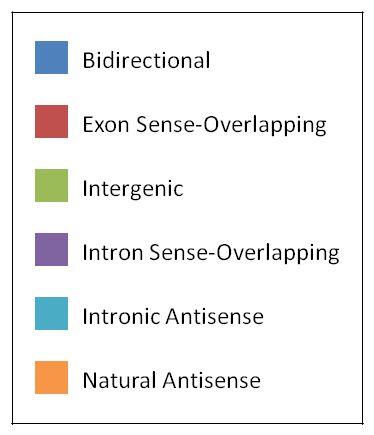
**

**Supplementary Figure 2. qRT- PCR Analysis of select lncRNAs.** Five lncRNAs that were deteceted as differentially expressed between patients #5308 and #4670 by microarray were confirmed by qRT-PCR (with the same directional change).

**
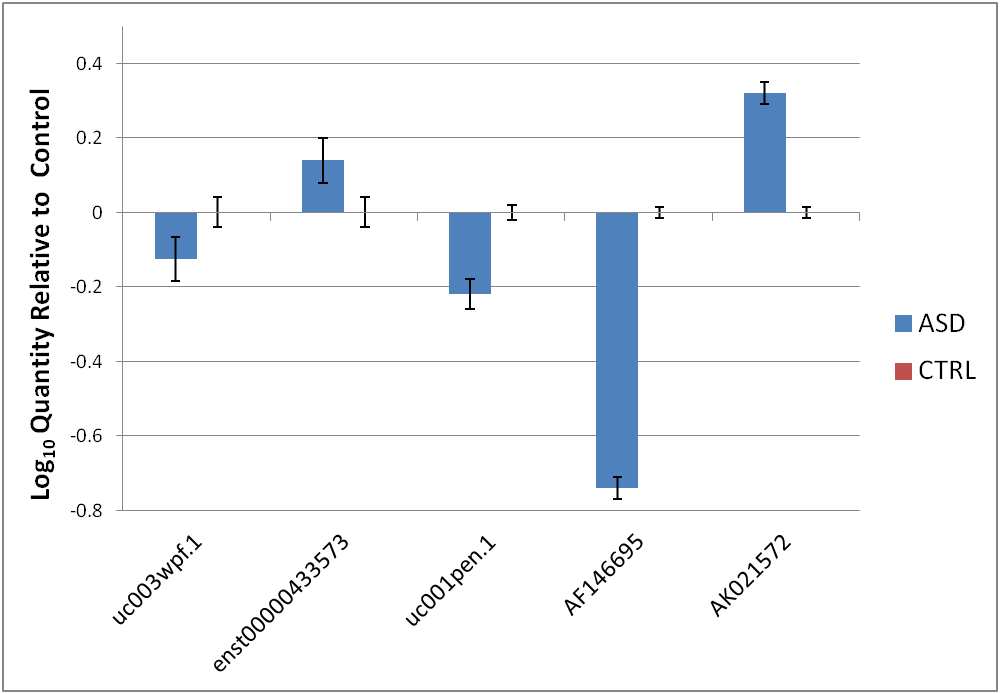
**

**Supplementary Figure 3. Relative orientation and distance to the nearest transcriptional start site (TSS) of all differentially expressed lncRNAs.**

**
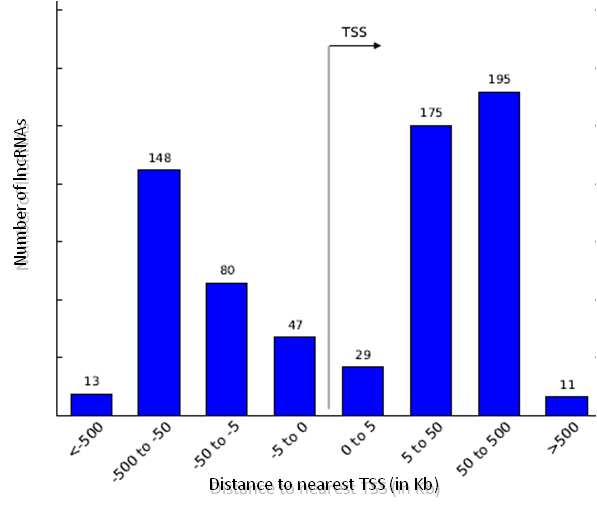
**

Supplementary Tables

**Supplementary Table 1.** Characteristics of patients from whom brain samples were obtained.

| **UMB#** | **Sex** | **Race** | **Diagnosis** | **Age (yrs)** | **Age (days)** | **PMI (hrs)** | **RIN** | **Cause of death** |
| --- | --- | --- | --- | --- | --- | --- | --- | --- |
| 5308 | Male | Caucasian | Autism (ADI-R) | 4 | 182 | 21 | 8.9-9.1 | Skull fracture |
| 5302 | Male | Caucasian | Autism (Clinical) | 16 | 119 | 20 | 8.8-8.9 | DKA |
| 4670 | Male | Caucasian | Control | 4 | 237 | 17 | 8.9-9.0 | Commotio Cordis |
| 4848 | Male | Caucasian | Control | 16 | 271 | 15 | 9.0-9.0 | Drowning |

RIN = RNA integrity number calculated from isolated RNA used for analysis; ADI-R = Autism Diagnostic Interview-Revised; PMI = Post-mortem interval

**Supplementary Table 2.** Source of lncRNAs contained on ArrayStar lncRNA microarray v2.0.

| **Database** | **# of lncRNAs** | **Literature Source** | **# of lncRNAs** |
| --- | --- | --- | --- |
| RefSeq (March 2011) | 2,608 | Khalil *et al^1^* “lincRNAs” | 3,289 |
| UCSC Known Genes 4 | 10,380 | Calin *et al^2^* “T-UC RNAs” | 962 |
| Ensembl 37.59 | 23,383 | Rinn *et al^3^* Hox cluster ncRNA | 407 |
| H-invDB 7.0 | 2,568 | Orom *et al*^4^ enhancer lncRNAs | 3,019 |
| RNAdb 2.0 | 1,492 |  |  |
| NRED (March 2011) | 1,112 |  |  |

1. Khalil AM, Guttman M, Huarte M, Garber M, Raj A, Rivea Morales D, *et al* (2009). *Proc Natl Acad Sci USA* **28**:11667-11672.

2. Calin GA, Liu CG, Ferracin M, Hyslop T, Spizzo R, Sevignani C, *et al* (2007). *Cancer Cell* **3**:215-229.

3. Rinn JL, Kertesz M, Wang JK, Squazzo SL, Xu X, Brugmann SA, *et al* (2007). *Cell* **7**:1311-1323.

4. Orom UA, Derrien T, Beringer M, Gumireddy K, Gardini A, Bussotti G, *et al* (2010). *Cell* **1**:46-58.

**Supplementary Table 3.** lncRNAs selected for qRT-PCR confirmation and the primers used.

| **Sequence Name** | **Source** | **Forward Primer** | **Reverse Primer** |
| --- | --- | --- | --- |
| uc003wpf.1 | UCSC Knowngene | GGGAGGGCCCAGCAAAACCC | CTGTGGGGCTGGACGGGAGA |
| ENST00000433573 | Ensembl | TGCATACCCAACTCAACCTG | ATGCATCAGGGCAGTAGGAA |
| uc001pen.1 | UCSC Knowngene | TGCCAGTTTTTCTGCTAGGTCCTG | CAGCCTCGGAGCTGCTGTGG |
| AF146695 | Misc RNA | AGGGTCATGGTGAGATGGAG | CAGCCCATACCAGTGCTTTT |
| AK021572 | Misc RNA | TGCCATATCACCTCTTGCAG | ACATGGCCACTGCTCTCTCT |

**Supplementary Table 4.** Genes near differentially expressed lncRNAs that were previously implicated in ASD or shown to be differentially expressed in ASD brains.

| **Cataloged in**  **AutDB or AGD** | **Differentially Expressed in ASD brains in Voineagu *et al* study** | |
| --- | --- | --- |
| DHCR7 | DACH1 | KIAA0427 |
| DLGAP2 | DUSP5 | LGALS3 |
| DRD3 | ECE2 | MAPRE2 |
| HLA-A | FBLN2 | NTSR2 |
| RPL10 | GSTT1 | PFKP |
| SDC2 | HLA-A | TNRC6A |
| SHANK2 | HLA-H | TXNIP |
| UBE3A | IGFBPL1 |  |
| APBA2 | ITPR1 |  |
| BDNF | KCNB1 |  |
| DLX6 | KCNG1 |  |

AutDB^5^ and AGD^6^ (Autism Genetics Database) are curated online catalogs of autism susceptibility genes based on published findings

5. Basu SN, Kollu R, Banerjee-Basu S (2009). *Nucleic Acids Res* **37**:D832-836.

6. Matuszek G, Talebizadeh Z (2009). *BMC Med Genet* **10**:102.

**Supplementary Table 5.** Gene ontology analysis for differentially expressed mRNAs between autism and control prefrontal cortex.

| Category | Term | P-Value | Bonferroni | Benjamini | FDR |
| --- | --- | --- | --- | --- | --- |
| SP_PIR_KEYWORDS | alternative splicing | 1.70E-04 | 3.90E-02 | 3.90E-02 | 2.10E-01 |

**Supplementary Table 6.** Gene ontology analysis for differentially expressed mRNAs within control prefrontal cortex versus cerebellum.

| Category | Term | P-Value | Bonferroni | Benjamini | FDR |
| --- | --- | --- | --- | --- | --- |
| GOTERM_BP_FAT | synaptic transmission | 1.70E-10 | 6.40E-07 | 6.40E-07 | 3.10E-07 |
| GOTERM_BP_FAT | transmission of nerve impulse | 2.60E-09 | 9.90E-06 | 4.90E-06 | 4.90E-06 |
| GOTERM_CC_FAT | neuron projection | 1.00E-08 | 5.80E-06 | 5.80E-06 | 1.50E-05 |
| SP_PIR_KEYWORDS | alternative splicing | 1.20E-07 | 8.00E-05 | 8.00E-05 | 1.80E-04 |
| SP_PIR_KEYWORDS | cleavage on pair of basic residues | 2.20E-07 | 1.50E-04 | 7.40E-05 | 3.20E-04 |
| UP_SEQ_FEATURE | splice variant | 2.20E-07 | 9.50E-04 | 9.50E-04 | 4.10E-04 |
| GOTERM_BP_FAT | cell-cell signaling | 6.60E-07 | 2.50E-03 | 8.20E-04 | 1.20E-03 |
| GOTERM_CC_FAT | synapse | 2.30E-06 | 1.30E-03 | 6.40E-04 | 3.30E-03 |
| GOTERM_MF_FAT | substrate specific channel activity | 2.10E-06 | 2.50E-03 | 2.50E-03 | 3.30E-03 |
| GOTERM_CC_FAT | postsynaptic membrane | 2.50E-06 | 1.40E-03 | 4.70E-04 | 3.60E-03 |
| SP_PIR_KEYWORDS | postsynaptic cell membrane | 3.90E-06 | 2.70E-03 | 9.00E-04 | 5.90E-03 |
| GOTERM_CC_FAT | dendrite | 4.10E-06 | 2.40E-03 | 5.90E-04 | 6.10E-03 |
| SP_PIR_KEYWORDS | developmental protein | 4.40E-06 | 3.00E-03 | 7.60E-04 | 6.60E-03 |
| GOTERM_MF_FAT | calcium ion binding | 4.80E-06 | 5.90E-03 | 2.90E-03 | 7.80E-03 |
| GOTERM_CC_FAT | synapse part | 6.70E-06 | 3.80E-03 | 7.70E-04 | 9.80E-03 |
| GOTERM_MF_FAT | channel activity | 7.30E-06 | 8.90E-03 | 3.00E-03 | 1.20E-02 |
| GOTERM_MF_FAT | passive transmembrane transporter activity | 8.00E-06 | 9.70E-03 | 2.40E-03 | 1.30E-02 |
| SP_PIR_KEYWORDS | synapse | 9.80E-06 | 6.70E-03 | 1.40E-03 | 1.50E-02 |
| SP_PIR_KEYWORDS | calcium | 1.20E-05 | 7.90E-03 | 1.30E-03 | 1.70E-02 |
| GOTERM_BP_FAT | regulation of nervous system development | 1.10E-05 | 3.90E-02 | 9.90E-03 | 2.00E-02 |
| SP_PIR_KEYWORDS | amidation | 1.70E-05 | 1.20E-02 | 1.70E-03 | 2.50E-02 |
| GOTERM_BP_FAT | regulation of neuron projection development | 1.60E-05 | 6.00E-02 | 1.20E-02 | 3.00E-02 |
